# Development and Evaluation of a Virtual Research Environment to Improve Quality of Care in Overcrowded Emergency Departments: Observational Study

**DOI:** 10.2196/13993

**Published:** 2019-08-08

**Authors:** Charles-Henri Houze-Cerfon, Christine Vaissié, Laurent Gout, Bruno Bastiani, Sandrine Charpentier, Dominique Lauque

**Affiliations:** 1 Emergency Department Toulouse University Hospital Toulouse France; 2 Institut Toulousain de Simulation en Santé Toulouse France; 3 UMR Education, Formation, Travail, Savoir University Toulouse 2 Jean Jaures Maison de la Recherche Toulouse France; 4 Emergency Department Albi University Hospital Albi France

**Keywords:** virtual reality, interprofessional relations, emergency medicine

## Abstract

**Background:**

Despite a wide range of literature on emergency department (ED) overcrowding, scientific knowledge on emergency physicians’ cognitive processes coping with overcrowding is limited.

**Objective:**

This study aimed to develop and evaluate a virtual research environment that will allow us to study the effect of physicians’ strategies and behaviors on quality of care in the context of ED overcrowding.

**Methods:**

A simulation-based observational study was conducted over two stages: the development of a simulation model and its evaluation. A research environment in emergency medicine combining virtual reality and simulated patients was designed and developed. Afterwards, 12 emergency physicians took part in simulation scenarios and had to manage 13 patients during a 2-hour period. The study outcome was the authenticity of the environment through realism, consistency, and mastering. The realism was the resemblance perceived by the participants between virtual and real ED. The consistency of the scenario and the participants’ mastering of the environment was expected for 90% (12/13) of the participants.

**Results:**

The virtual ED was considered realistic with no significant difference from the real world with respect to facilities and resources, except for the length of time of procedures that was perceived to be shorter. A total of 100% (13/13) of participants deemed that patient information, decision making, and managing patient flow were similar to real clinical practice. The virtual environment was well-mastered by all participants over the course of the scenarios.

**Conclusions:**

The new simulation tool, Virtual Research Environment in Emergency Medicine, has been successfully designed and developed. It has been assessed as perfectly authentic by emergency physicians compared with real EDs and thus offers another way to study human factors, quality of care, and patient safety in the context of ED overcrowding.

## Introduction

### Background

Analysis of medical errors shows that among human factors, psychological, cognitive, and organizational features are directly related to the quality of care. Emergency department (ED) overcrowding and patient boarding result in delays in care, increased short-term mortality, and worsened patient experiences and safety [1,2]. Emergency physicians have to deal with several patients in a short period of time and anticipate risk of errors linked to interruptions and disruptions [3]. To alleviate the problem of ED overcrowding, various conceptual models of care have been invented by researchers, which mainly focus on organizational strategies and input-throughput-output processes [4-7]. However, few have analyzed the cognitive processes used by emergency physicians coping with overcrowding, or the impact of overcrowding on team processes [8]. Team processes are defined as the cognitive, verbal, and behavioral activities of a team working together. They include the nontechnical skills for crisis resource management (CRM), such as leadership, teamwork, situation awareness, task management, and decision making. In a systematic literature review, Schmutz has shown that valid measures and adequate tools are required for investigating the effects of team processes on clinical performance and safety [9].

Team process behavior (eg, how the team functions and whether the team and its members grow, develop, and improve over time) is influenced by patient volume, the number and attitude of other caregivers, as well as changes in the department’s organization [7]. Variations of these confounding factors make it difficult to analyze the direct relationship between overcrowding, team processes, and quality of care. Simulation in a virtual environment could expose emergency physicians to flow variation only and show its impact on CRM skills and quality of care (test relevance, working diagnosis, decision-making time, and patient orientation) [10]. Online virtual worlds such as serious games have shown their effectiveness concerning medical education and training of teamwork skills, including leadership, coordination, and communication [11,12]. They offer a safe and controlled setting without real patients’ risks and issues.

To our knowledge, no study has yet been conducted on the development and evaluation of a virtual ED related to quality care.

### Objectives

The goal of this study was to develop a virtual ED intended to replicate real patient flow management by emergency physicians and evaluate the authenticity of this new simulated research model.

## Methods

### Study Design

This simulation-based observational study was conducted in two stages. The first stage was the design and development of a virtual environment in emergency medicine. The second stage was an observational study to evaluate this environment.

### Design and Development of the Virtual Environment

**Figure 1:**
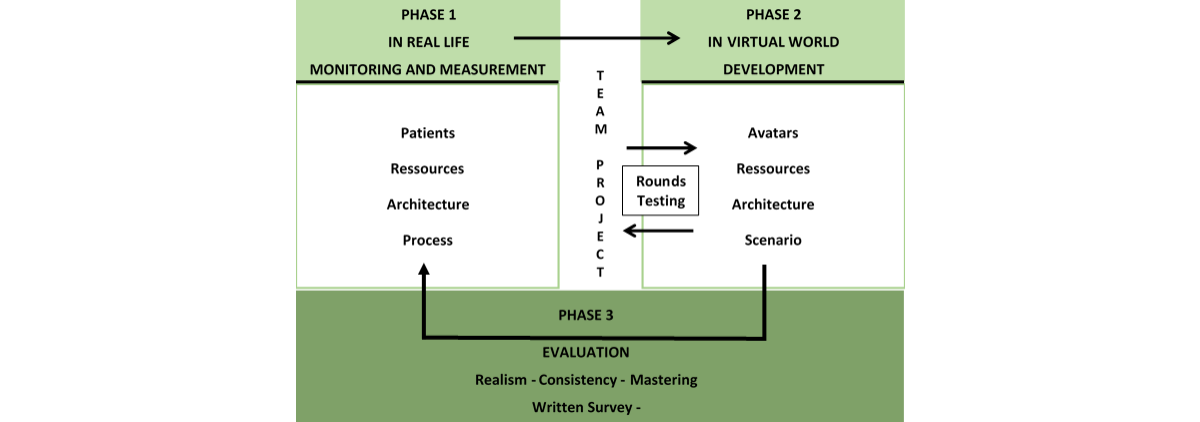
Design, development, and assessment of Virtual Research Environment in Emergency Medicine.

Virtual Research Environment in Emergency Medicine (VIREM) is a hybrid simulation that combines a virtual environment (virtual multiplayer world), technical skills simulation, and simulated patients (Figure 1) . Second life^®^ was used as virtual world online because it is an interactive, 3-dimensional, low-cost environment easily accessed by many participants who interact and communicate together (see Multimedia Appendix 1). Patients and caregivers were represented as characters called avatars that interact with their environment (see Multimedia Appendix 2).

A research team designed and developed the virtual ED. The team consisted of 3 emergency physicians and 1 nurse who had more than 10 years of experience in emergency medicine, a game developer, and an expert in virtual simulation didactics. They designed the simulated ED premises and equipment in accordance with the French Emergency Medicine Society’s guidelines on ED architecture and operation [13]. In a scenario, the virtual ED team included avatars for 1 emergency physician, 1 resident in postgraduate years 2, and 3 nurses for triage, ambulatory care, and nonambulatory care, respectively (see Multimedia Appendix 3). The emergency physician worked both as team leader and caregiver. The medical conditions of patient avatars were selected from a French regional registry of 37 ED populations based on the most prevalent conditions; the final choice of the 13 cases was based on team agreement as representative of the usual patients in an urban busy ED. The history, clinical signs, test results, and course were developed for each patient. Operational procedures were designed for the nurse avatars who could triage, draw blood samples, perform electrocardiograms, and deliver medications [12]. The median length of time of each procedure and intervals from test ordering to data-ready time were measured in patients admitted in 3 different EDs and reported in the scenario (Table 1).

The patient flow was set at a level higher than the recommended maximum with 4 patients per physician per hour to simulate overcrowding conditions. The virtual ED was developed and iteratively tested by the research team to correct bugs and improve patient flow, processes, and interactions between the avatars and their environment.

**Table 1:** Length of time of real emergency procedures used in Virtual Research Environment in Emergency Medicine.

Real emergency procedures (n)		Duration (min), median (interquartile range)
**Nurse 2**		
	Administrative reception time (20)	4 (2-4)
**Nurse 1 tasks**		
	Triage time (20)	4 (3-5)
	Time to prepare and give the medications (17)	5 (4-6)
	Electrocardiogram recording (16)	5 (4-5)
	Blood drawn to data-ready time (18)	88 (67-115)
	Specialty physician consultation, data-ready time (16)	45 (36-50)
**Resident tasks**		
	History and examination of patient (20)	14 (10-15)
	Perform cast (10)	15 (12-18)
	Sutures (10)	21 (13 -39)
	Medical records (20)	10 (8-12)
	Prescription (18)	7 (6-8)
	Patient information (16)	5 (5-5)
**Radiological test ordering to data-ready time**		
	X-ray (19)	40 (37-48)
	Computed tomography scan (11)	60 (60-120)
	Ultrasounds (15)	105 (102-108)
**Specialty physician consultation**		
	Data ready time (16)	45 (36-50)

### Selection of Participants for Virtual Research Environment in Emergency Medicine Evaluation

We recruited emergency physicians in 2 EDs of a multifacility academic hospital, which together treated more than 164,059 patients aged 15 years or older in 2017 [14]. They had at least 3 years’ experience in emergency medicine and were not involved with the research team. Upon enrollment, confidentiality was guaranteed, and all physicians gave their written informed consent. This study was carried out with the agreement of the ethics committee of the University and the French National Council for Information Technology and Liberties (Reference 19880021v0).

### Study Protocol

Data collection took place in the University Institute of Health Simulation. Data were collected by short structured interviews and questionnaires by a nonparticipant observer. The participants were alone in a room with a computer and a technical skill simulator to perform the sutures. They each had a 30-min briefing focused on the resources available, how to interact with the environment, and communicate with the patients and caregivers as a physician avatar. The participants interacted with the virtual ED by controlling their avatars and with a headset (Voice over Internet Protocol). They used a phone to call other caregivers or specialists who were out of reach. The scenario lasted 2 hours and was followed by a short, structured interview lasting 20 min. A total of 4 instructors from the research team were required to pilot, respectively, the patients, nurse 1 avatar, resident avatar, and nurse 2 avatars or other caregiver avatars (dispatcher, specialist). The instructors, according to their role, triggered the different procedures whose duration had already been defined (Table 1). Participants and instructors used Dell Precision T1700 computers, Nvidia GTX 970 type graphics card with 22.5“ full high-definition screens.

### Evaluation of the Environment

The study outcome was the authenticity perceived by participants working in this virtual ED. The perception of authenticity by users results from the fidelity of the environment (setting and available resources), the situations experienced, and the temporal fidelity of procedures [15]. VIREM was adjudicated as authentic if the 3 components, realism, consistency, and mastering, were validated.

The realism was defined as the resemblance of the virtual ED with a real ED. Physicians have been asked to evaluate their own ED and the virtual environment after the scenario on a 7-item questionnaire about facilities, resources available, and delays. Each question was scored according to a 5-point Likert scale from very dissatisfied to very satisfied. The virtual ED was deemed realistic if the satisfaction level of the 7 items was not different between the virtual and real ED.

The consistency of the proposed rules and situations was assessed during individual directive interviews performed after the scenario. Participants gave their opinion on the quality of information provided by the scenario, their ability to make decisions, and whether the scenario was proper to manage the patient flow and preserve the quality of care. The consistency of VIREM was deemed to be good if these 3 criteria were positive for at least 90% (12/13) of participants.

The mastering of the environment was assessed by an independent observer during the scenario. A total of 5 cognitive tasks were rated: (1) acceptance and participation in the scenario; (2) environment testing; (3) ability to make decisions; (4) anticipation; and (5) use of the environment, procedures, and resources. We considered that mastering the VIREM was good when more than 90% (12/13) of participants fulfilled these 5 criteria.

### Statistical Analysis

All data were entered into an Excel spreadsheet, (Microsoft Corp) and the statistical analysis was performed with Stata (Version 12.0, Stata Corp). Categorical data were presented as frequencies and percentages (%) and continuous data as mean with SDs or median with interquartile ranges if not normally distributed. The realism satisfaction score between the virtual ED and the real ED environment was compared using a Wilcoxon signed-rank test. Results with a *P*<.05 were considered significant.

## Results

### Stage 1: Virtual Emergency Department

The virtual ED was designed as a level 1 trauma center in a major adult referral hospital. It consisted of a reception and triage area, 5 examination rooms for monitoring with a computer, a medication preparation room, a waiting room, a medical office, and a radiology department with ultrasound and computed tomography scan equipment. The participants took up their shift when the scenario began. A total of 3 patients were already undergoing care in the examination rooms. Thereafter, 4 and 6 patients attended the ED during the first and second hours, respectively (Table 2).

During the scenario, the participant physician led his avatar and fully reviewed the patient’s history and complaints in the available examination room. He selected parts of the patient’s body for physical examination, prescribed biological tests, x-rays, and treatment and checked test results. He communicated by Voice over Internet Protocol with the instructor who led the patient avatars to explain procedures and care to patients, assess their pain, and discuss the case with other specialists. The participant could coordinate activities with team members, exchanging information, communicating, making decisions, keeping others aware of the situation, planning, and prioritizing the tasks. The participant could perform the task himself or ask the resident or nurse avatar to do it (Table 1).

A total of 12 enrolled emergency physicians completed the study. The sex ratio was 1:4, median age 30 years (range 29-35), and median experience in emergency medicine as a resident and attending physician was 4 years (range 3-10; Table 3). All participants used software and networks at work and had basic computer skills. Only 1 participant was familiar with the virtual environment.

**Table 2:** Screenplay of Virtual Research Environment in Emergency Medicine.

Min	Instructor 1: Resident^a^	Instructor 2: Nurse 1/dispatcher/other caregivers^a^	Instructor 3: Nurse 2/patients (P)^a^
T-1	—^b^	—	P1 —> room 1/P2—> room 2
T+0	Physician 1	—	—
T+5	—	Physician family calls	—
T+10	—	—	P3 —> ED^c^ hall
T+14	—	—	P3 —> triage room
T+15	—	Radiologist calls for P2	—
T+19	—	—	P3 —> room 3a and call EP^d^
T+21	—	—	P4/P13 —> ED hall
T+24	—	—	P4 —> waiting room
T+25	—	—	P13 —> triage room
T+30	MD (gastro) calls for P1	—	—
T+35	—	Gastroenterologist calls	P13 —> room 2b
T+36	—	—	Call nurse 1 for IV line P13
T+37	—	—	P4 —> triage room
T+42	—	IV^e^ line P13	Call EP: “where do I install P4”
T+43	—	—	P5 —> ED hall
T+44	—	Dispatcher calls EP	—
T+46	—	—	P5 —> triage room
T+50	—	Nurse calls EP: “blood test sent”	—
T+51	—	—	P5 —> room 3b
T+52	—	Nurse calls EP: “what is the blood test for p5?”	—
T+62	—	—	P6 —> ED hall
T+66	—	—	P6 —> triage room
T+70	—	—	P7 —> ED hall
T+71	—	—	P6 —> room available or call EP
T+72	—	Dispatcher calls EP	—
T+74	—	—	P7 —> triage room
T+78	Call EP: “neurologist is ready”	—	—
T+79	—	—	P7 —> room available or call EP
T+81	—	—	P8 —> ED hall
T+84	—	—	P8 —> triage room
T+89	—	—	P8 —> room available or call EP
T+90	Call EP: “P13 have a Troponin at 45”	—	—
T+91	—	—	P9 —> ED hall
T+95	—	—	P9 —> triage room
T+96	—	—	P10 —> ED hall
T+99	—	—	P9 —> room available or call EP
T+101	—	—	P10 —> triage room
T+105	—	—	P11 —> ED hall
T+106	—	—	P10 —> room available or call EP
T+110	—	—	P11 —> triage room
T+111	—	—	P12 —> ED hall
T+114	—	—	P11 —> room available or call EP
T+115	—	—	P12 —> triage room

^a^Each column describes the actions carried out by the 3 instructors according to their function in the scenario. The actions are displayed chronologically after the beginning of the scenario. For example, at the 42nd min the second instructor who controls nurse 1 must place an IV line to patient 13, and the third instructor who controls nurse 2 calls the emergency physician to ask him in which emergency room patient 4 would be installed.

^b^No action.

^c^ED: emergency department.

^d^EP: emergency physician.

^e^IV: intravenous.

**Table 3:** Characteristics of the participants (n=12).

Characteristics		Value
Age (years), median (IQR^a^)		30 (29-35)
Males, n (%)		7 (58)
Experience in emergency medicine (years), median (IQR)		4 (3-10)
Basic level of computer experience^b^, n (%)		12 (100)
**Rate of playing computer games^c^, n (%)**		
	Never	10 (83)
	Occasionally	2 (16)
First time with experimental virtual reality system, n (%)		11 (91)
Basic level of knowledge on 3D images production^b^, n (%)		12 (100)
**Level of knowledge on virtual reality^b^, n (%)**		
	None	11 (91)
	Basic	1 (8)

^a^IQR: interquartile range.

^b^Items were graded on Likert-type scale (1=none, 2=basic, 3=intermediate, 4=expert).

^c^Items were graded on Likert-type scale (1=never, 2=occasionally, 3=less than 50% days, 4=more than 50% days, 5=every day).

### Stage 2: Evaluation of Authenticity

#### Realism

Participant satisfaction with the facilities (premise and examination room) and resources (team members) was similar in the virtual ED and in their real ED (Table 4).

The participants considered the timeliness of procedures in the virtual ED satisfactory, and they perceived the lengths of time shorter. Therefore, our virtual ED was realistic for facilities and resources but not for procedures, although lengths of time used in the virtual ED were based on real-life data.

**Table 4:** Evaluation of the realism of the virtual emergency department versus the real environment (n=12).

Categories		Satisfaction, median (interquartile range)		*P* value^a^
		Real environment, range 1-5^b^	Virtual environment, range 1-5^b^	
**Facilities**				
	Premises	3 (3-4)	4 (3-4)	.41
	Examination rooms	3 (3-4)	3 (3-4)	.96
**Procedures**				
	Duration of blood drawing and electrocardiogram	4 (3.6-4)	4 (4-5)	.04^c^
	Blood drawn or radiological test ordering to data-ready time	3 (2.7-3)	3.5 (3-4)	.008^c^
	Specialty physician consultation ordering to data-ready time	2 (2-3)	3 (3-3.2)	.003^c^
**Resources**				
	Number of nurses	3 (3-3)	3 (2.7-3)	.08
	Number of residents	3 (3-4)	3 (3-3.2)	.32

^a^Wilcoxon signed rank test.

^b^Items were graded on Likert-type scale (1: very dissatisfied, 2: dissatisfied, 3: neither, 4: satisfied, 5: very satisfied).

^c^Significant difference.

#### Consistency

The virtual ED was considered consistent because all participants felt that patient information was consistent with reality and allowed for medical decisions similar to their clinical practice (100%). Strategies for managing patient flow and preserving quality of care in the context of overcrowding was deemed by all participants to be consistent with those used in their real ED (100%).

#### Mastering

All participants (N=13) performed the cognitive tasks assigned by the scenario, thus reflecting their mastery of this virtual environment. They actively participated in the simulation (100%), used the virtual environment (100%), made decisions (100%), anticipated actions (100%), and used the environment, procedures, and resources (100%).

## Discussion

### Principal Findings

This was the first study to propose a method for developing and validating a simulated research environment combining different simulation techniques as procedural simulation and virtual simulation [16]. Virtual environments are usually targeted toward the acquisition of skills and knowledge [11,12,17]. Our goal was to build an authentic research environment to study the cognitive strategies and CRM skills of physicians in a situation of high cognitive load because of patient flow in an ED. We relied on the definition of authenticity given by Petraglia: “Authenticity is not an intrinsic property of an object, but a judgment, a decision on the part of the user from the point of view of his or her past experiences and the socio-cultural context. Authentic virtual simulation is a game perceived as authentic by the learners” [15].

Our approach designed and assessed the virtual environment in the 3 dimensions of authenticity: realism, consistency, and mastering.

All participants found that the virtual environment was realistic and easy to master. A previous research study showed that knowledge of virtual reality was related to engagement. More knowledge about virtual reality leads to a greater sense of engagement [18]. Although the population in our study had a moderate level of computer experience and lacked knowledge about virtual reality, the physicians were effectively able to perform the cognitive tasks and get engaged in the simulation. This finding could be explained by the easy use of this virtual environment that does not require users to have specific skills or characteristics.

The realism of the premises and resources was validated by all participants. Perception of time for physician and nurse tasks, as well as evaluation of delays for test or consultation results were different in the scenario than in real life. All participants agreed that virtual care seemed shorter than real life one. This difference of perception between virtual and real ED has been explained by Block et al who found that duration judgments are affected by cognitive load. If participants are aware that duration judgments must be made (prospective paradigm), a greater cognitive load decreases the perception of duration [19]. Therefore, subjective assessment of procedure duration is not an accurate criterion of realism but instead may be an indicator of cognitive overload caused by patient flow in this scenario.

All participants agreed that VIREM was a valuable research environment for studying the cognitive processes of emergency physicians and the effect of patient flow on quality of care. Observation of participants’ attitudes during the test showed that they rapidly mastered this virtual environment. As a preliminary observation, we noticed that the 12 emergency physicians used different attitudes and strategies for managing the patient flow.

Flowerdew et al have shown that workload management is a complex skill that covers a wide range of behaviors [8]. This systemic review identified a link among workload management, safety, and error. For example, diagnostic errors increase with work overload.

As it has been suggested by Leblanc et al, a simulation model such as VIREM could be used to identify the safest workload management [20]. In addition, its accessibility with distributed teams and its low cost with only an up-to-date computer and internet connection would permit widespread studies to be conducted.

One of the main aspects of VIREM is that it also allows ED management training. The learnings from the implementation of the emergency response highlight the need for adapting a holistic approach by using core competencies in the domain of emergency management, such as preparedness, response system, patient care, and resource management (human and material). Most competencies can be obtained through traditional accredited education and training programs; on the other hand, education for personnel operating in emergency situations should be based on the acquisition of task-related, profession-specific, and cross-disciplinary competencies that cannot be easily acquired through those programs. VIREM will allow us to standardize competency-based education in emergency and resource management.

Similar to VIREM, other virtual environments could easily be designed for any health organization that would be interested in analyzing the links among human factors, diseases, resources available to health professionals in their workplaces, and quality of care. Next, it could also be a good way to deliver an effective and up-to-date interprofessional training in many countries and more particularly in low and medium income countries where access to training is a challenge for financial, geographical, political, and/or institutional reasons. Afterwards, it could facilitate the implementation of interprofessional training for health workers in even the remotest regions.

### Limitations

The major limitation of this study was the sample size. Although only 12 physicians took part in the observational study, the duration of the simulation session was too long to involve more participants from the same hospital.

Second, we did not measure the participant stress level during the scenario. Simulations can induce stress that may influence decision making and performance [21,22]. Also, the perception of nonreal scenario is likely to alter the alertness and stress level of the participant, which is unlikely to be the same as the real-life situation. Future studies using VIREM will have to integrate the stress level, which affects emotion, perception, memory, judgment, and reasoning [23]. Third, the physical activity–induced fatigue for emergency physicians working in a real ED was not reproducible in this virtual environment. Another potential limitation of the virtual environment would be the requirement for concentration and energy. The simulation would be less stressful as well. However, working in a real ED during a period of time as short as 2 hours presumably would not cause enough fatigue to influence decision making and behavior.

### Conclusions

We developed a new virtual environment, VIREM, to study strategies set in place by emergency physicians to manage patient flow in EDs. This virtual environment was assessed as authentic by emergency physicians and thus, offers a new research tool to study CRM skills and improve quality of care and patient safety in the context of ED crowding. Its use could be extended to a different geographical, sociological, and economic context, but further studies will be needed to validate it.

## Appendix

Multimedia Appendix 1 Multiview of Virtual Research Environment in Emergency Medicine.

Multimedia Appendix 2 Staff in Virtual Research Environment in Emergency Medicine.

Multimedia Appendix 3 Emergency room in Virtual Research Environment in Emergency Medicine.

## Abbreviations

CRM: crisis resource management
ED: emergency department
VIREM: Virtual Research Environment in Emergency Medicine
