# Supplementary material for: Development and Evaluation of a Virtual Research Environment to Improve Quality of Care in Overcrowded Emergency Departments: Observational Study
Source: JMIR Serious Games. 2019 Aug 8;7(3):e13993. doi: 10.2196/13993 (PMC6705008; doi:10.2196/13993)
Supplement: Multimedia Appendix 1 [file games_v7i3e13993_app1.pdf]

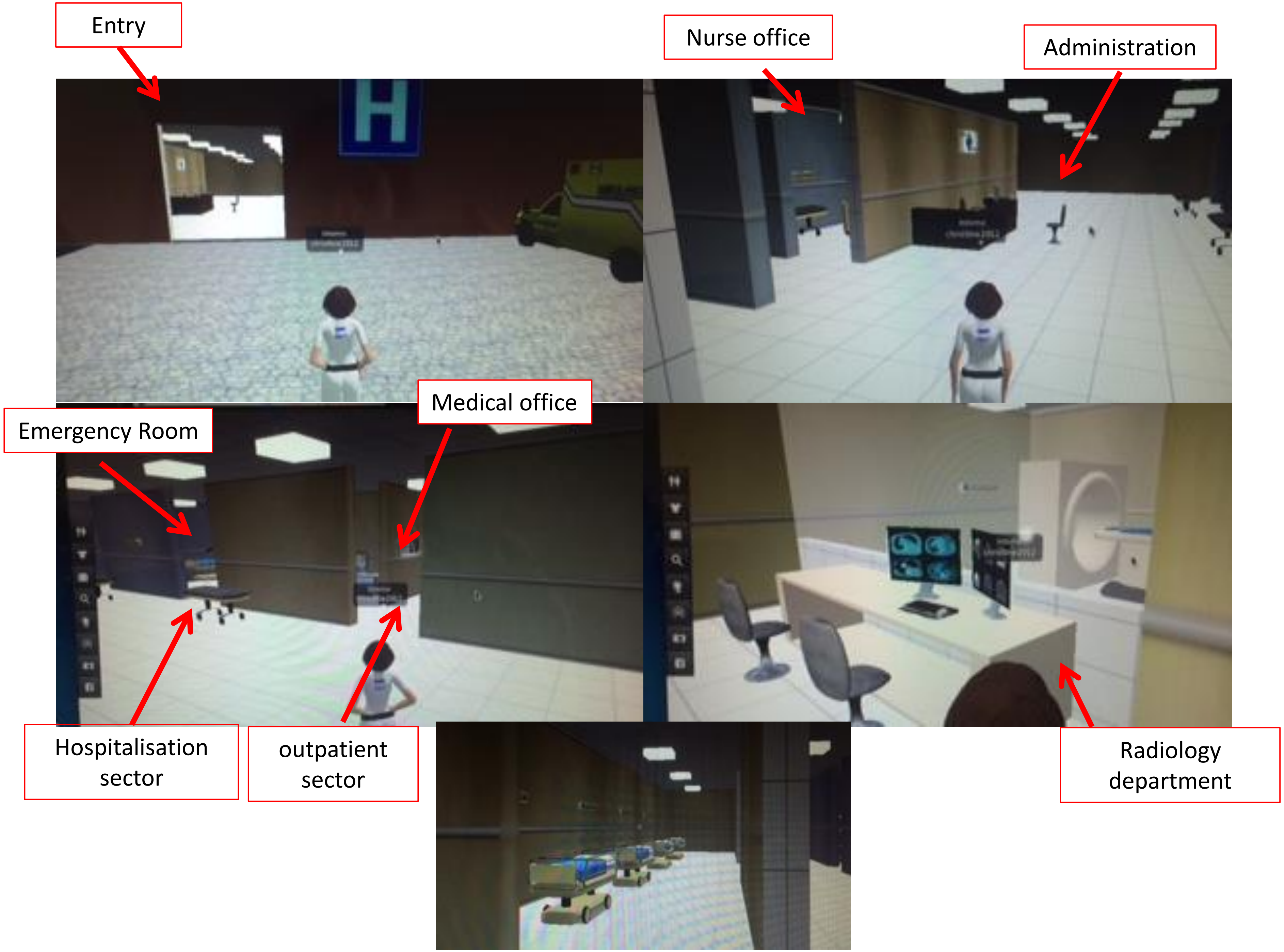
Entry
Nurse office
Administration
Emergency Room
Medical office
Hospitalisation sector
outpatient sector
Radiology department
