# Supplementary figures and images for: Development and Evaluation of a Virtual Research Environment to Improve Quality of Care in Overcrowded Emergency Departments: Observational Study
Source: JMIR Serious Games. 2019 Aug 8;7(3):e13993. doi: 10.2196/13993 (PMC6705008; doi:10.2196/13993)

# Emergency Room

Emergency Physician

Nurse

Patient

Resident

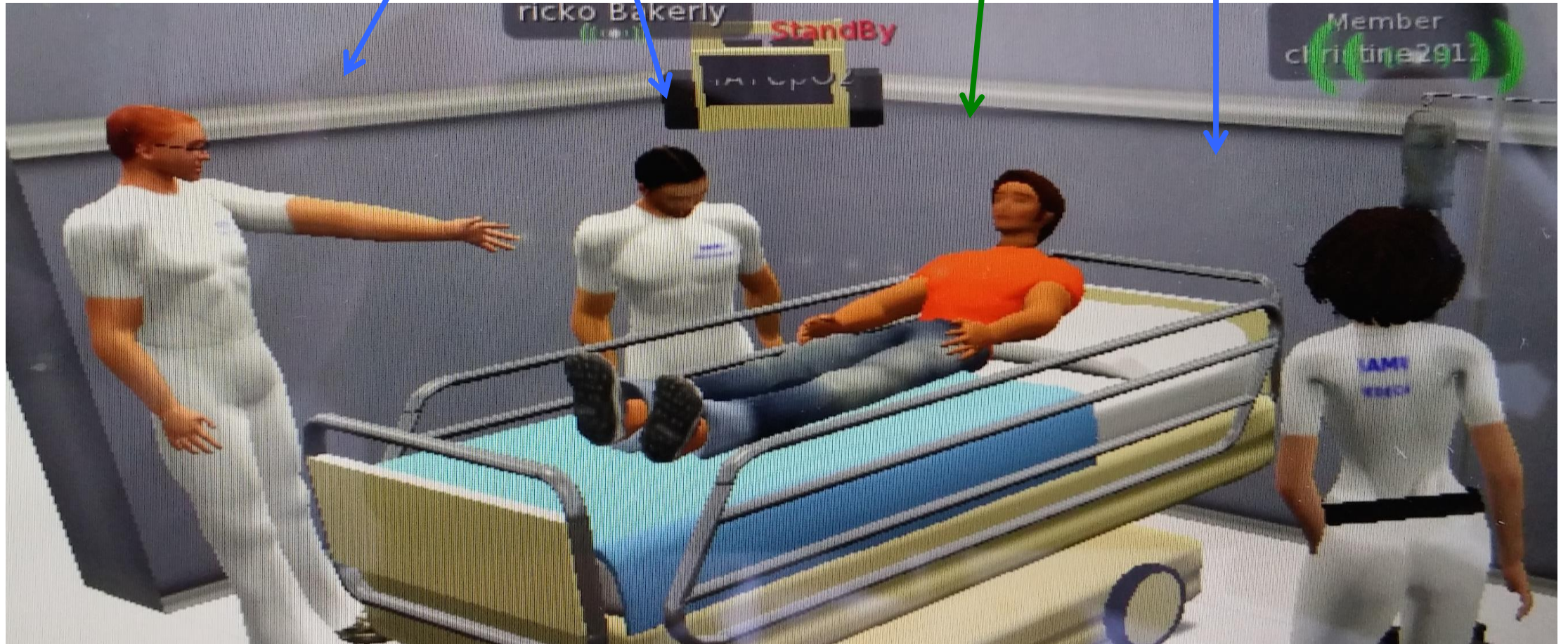

Supplement: Multimedia Appendix 2 [file games_v7i3e13993_app2.pdf]

Medical Office

Patient

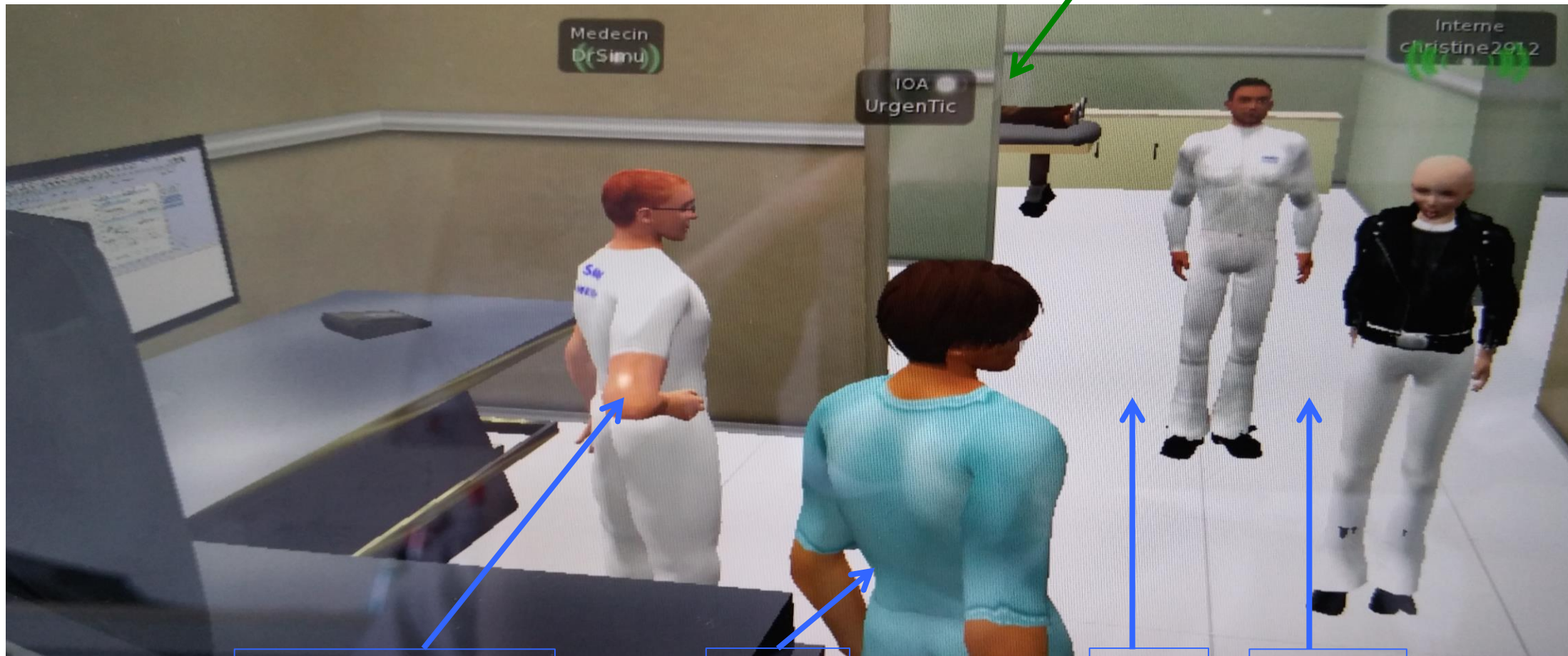

Emergency physician

Nurse

Nurse

Resident

Supplement: Multimedia Appendix 3 [file games_v7i3e13993_app3.pdf]
